# Recent biomedical advancements in graphene oxide- and reduced graphene oxide-based nanocomposite nanocarriers

**DOI:** 10.1186/s40824-022-00313-2

**Published:** 2022-11-26

**Authors:** Naline Bellier, Phornsawat Baipaywad, Naeun Ryu, Jae Young Lee, Hansoo Park

**Affiliations:** 1 School of Integrative Engineering, Chung-Ang University, Seoul, 06974 Republic of Korea; 2 Biomedical Engineering Institute, Chiang Mai University, Chiang Mai, 50200 Thailand; 3 School of Materials Science and Engineering, Gwangju Institute of Science and Technology, Gwangju, 61005 Republic of Korea

**Keywords:** Delivery, Functionalization, Nanomedicine, Drug loading, Therapeutic biomolecules

## Abstract

Recently, nanocarriers, including micelles, polymers, carbon-based materials, liposomes, and other substances, have been developed for efficient delivery of drugs, nucleotides, and biomolecules. This review focuses on graphene oxide (GO) and reduced graphene oxide (rGO) as active components in nanocarriers, because their chemical structures and easy functionalization can be valuable assets for in vitro and in vivo delivery. Herein, we describe the preparation, structure, and functionalization of GO and rGO. Additionally, their important properties to function as nanocarriers are presented, including their molecular interactions with various compounds, near-infrared light adsorption, and biocompatibility. Subsequently, their mechanisms and the most appealing examples of their delivery applications are summarized. Overall, GO- and rGO-based nanocomposites show great promise as multipurpose nanocarriers owing to their various potential applications in drug and gene delivery, phototherapy, bioimaging, biosensing, tissue engineering, and as antibacterial agents.

## Introduction

Graphene oxide (GO) is a two-dimensional (2D) nanomaterial comprising single-layer sheets of sp^2^ hybridized carbons, sites of sp^3^ hybridized carbons, and oxygenated groups, obtained from the oxidation and exfoliation of graphite [1]. First synthesized by British chemist B.C. Brodie in 1859, GO is obtained by chemical treatment of graphite flakes using strong oxidizers followed by dispersion and exfoliation in acidic mediums, a more refined method of which are commonly used today despite the production of resultant toxic gases [2–5]. However, current research in GO synthesis focuses on more cost-effective and eco-friendly development methods because interest in various applications of GO has increased owing to its attractive chemical and physical characteristics.

**Fig. 1:**
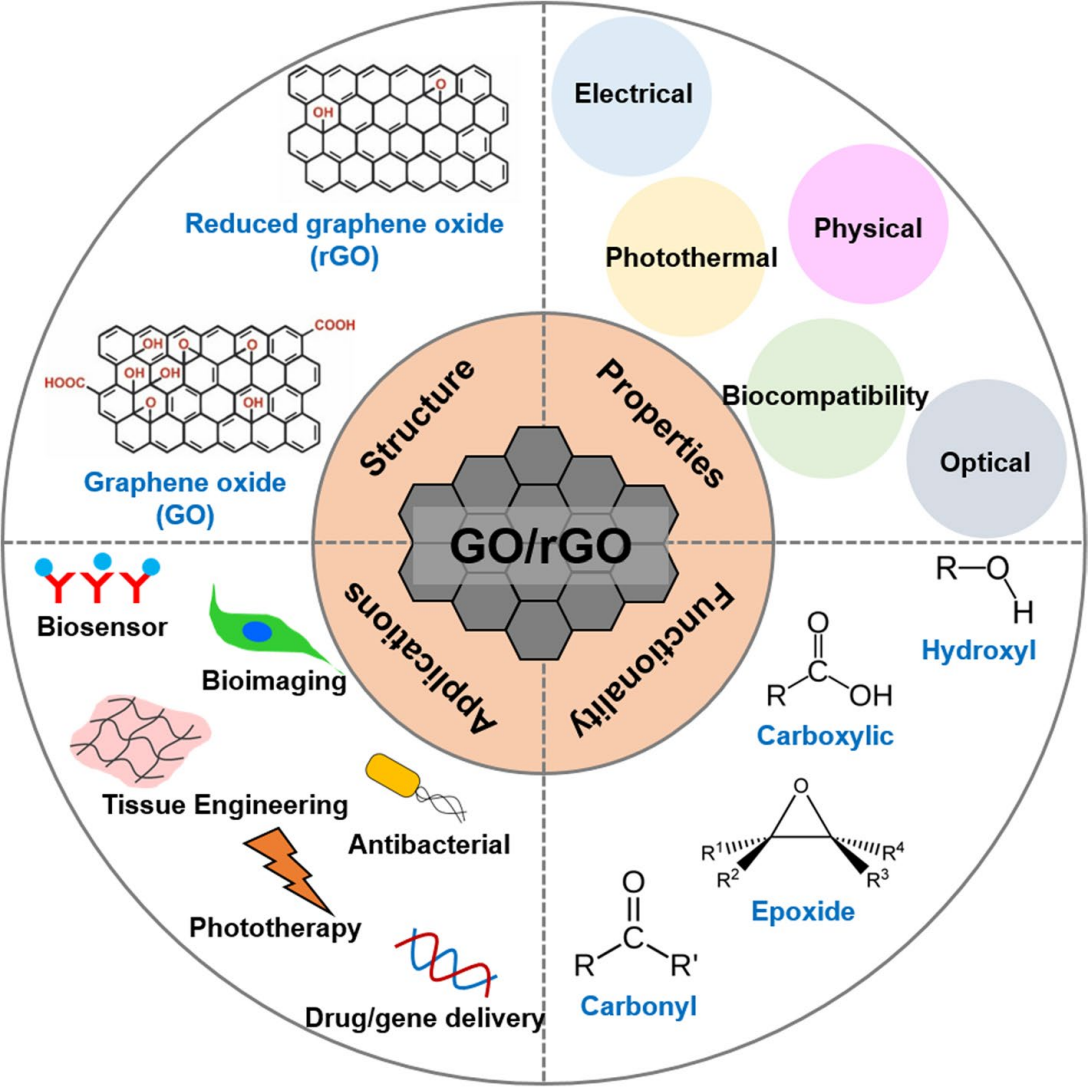
Graphical summary of recent biomedical advancements in graphene oxide- and reduced graphene oxide-based nanocomposite nanocarriers

GO is hydrophilic and highly dispersible in water and polar organic solvents because of its oxygen-containing functionalities, such as hydroxyl, carboxyl, carbonyl, epoxide, phenol, lactone, and quinone groups [6–8]. Carboxylic groups are located on the edges of GO, whereas epoxide and hydroxyl groups are present on the basal plane of GO [9–11]. Furthermore, GO exhibits excellent and unique properties, including a 2D planar structure, large surface area, straightforward modification, chemical stability, good biocompatibility, and high mechanical strength [8, 12, 13]. In particular, GO can strongly interact with various small molecules and macromolecules (e.g. drugs, proteins, metals, biomolecules, and cells) via π-π stacking, covalent bonding, hydrophobic interactions, electrostatic forces, and hydrogen bonding [6, 13, 14]. Because of such unique characteristics, GO has great potential in nanomedicine and biomedical applications which are presented in Fig. 1 [14].

Chemical reduction of GO is the most widely applied method for preparing reduced GO (rGO) [15]. Various chemical reductants, such as anhydrous hydrazine [16], hydrazine monohydrate, L-ascorbic acid, sodium borohydride [17], hydroquinone [18], birch [19], glucose [20], hydroxylamine [21], pyrrole [22], amino acids [23], strongly alkaline solutions [24], and urea [25] have been reported to remove the majority of oxygenated functional groups and partly restore sp^2^ carbon bonds in graphene [26–28]. Chemical reactions increase the conductivity, hydrophobicity, and π-π stacking interactions, which are important for drug delivery applications [15, 28]. Typically, hydrophobic anticancer drugs and small molecules can be loaded more efficiently onto rGO surfaces via π-π stacking and hydrophobic interactions compared to GO [17, 29]. Additionally, rGO nanosheets have been widely studied for phototherapy owing to their large surface area, high light-adsorption ability, and excellent photothermal effect [30–32]. Because of these exceptional properties, rGO has been extensively explored as a promising material for multi-purpose nanocarriers.

In addition to the physical and photothermal properties of GO and rGO, which allow for effective cancer treatment via drug and gene delivery and phototherapy, respectively, both materials have been widely explored for bioimaging, biosensing, tissue engineering, and antibacterial applications. This is because of other significant properties of GO/rGO, such as electrical conductivity, light absorbance and emission, and biological effects. Although other methods will be discussed, GO/rGO-based materials have been particularly popular in bioimaging because of their fluorescent emission under the right excitation wavelength [33, 34]. Meanwhile, GO-/rGO-based biosensors use their fluorescence quenching abilities [35, 36] although high electrical conductivity of rGO makes it a suitable candidate for electrochemical (EC) or electrochemiluminescence (ECL) assays [37, 38]. Furthermore, GO/rGO is known to promote stem cell proliferation and differentiation, which has encouraged research in their use in tissue engineering, particularly that of cardiac and nerve tissues, which improves in the presence of a conductive material [39–41]. Finally, GO/rGO is known to be cytotoxic towards bacteria, which has prompted research in antibacterial applications [42, 43].

Several review papers have focused on graphene and GO for biomedical applications; however, the discussion of rGO remains only a footnote in these [44–48] In this review, we provide a brief overview of the history and preparation of GO and rGO as well as their chemical structures, functionalization methods, and properties. Their mechanisms and applications in the form of nanocarriers in drug and gene delivery, phototherapy, bioimaging, biosensing, tissue engineering, and bacterial elimination, along with their potential as multipurpose nanocarriers, are also discussed.

## Synthesis and structure of graphene oxide and reduced graphene oxide

### Synthesis

British chemist B. C. Brodie first synthesized GO in the nineteenth century (1859) by treating graphite with a mixture of oxidizing agents (potassium chlorate (KClO_3_) and fuming nitric acid (HNO_3_)) [2]. After oxidative treatments with four repeated reactions, an increase in the overall mass of the graphite flakes was observed, which was believed to result from the presence of additional carbon, hydrogen, and oxygen atoms in the product [49]. Another common technique, modified from the Brodie method, was described by Stuadenmaier in 1898. The acidity of the mixture was increased using concentrated sulfuric acid (H_2_SO_4_) combined with fuming HNO_3_, followed by the addition of chlorate in multiple aliquots of KClO_3_ solution throughout the reaction [2, 50]. In 1957, chemists Hummers and Offeman developed another oxidation method [2, 3], a safer, quicker, and more efficient process where graphite reacts with a mixture of H_2_SO_4_, sodium nitrate, and potassium permanganate [51]. The difference from previous methods lies in the use of H_2_SO_4_ instead of HNO_3 _[50]. Altogether, all the methods mentioned above require extensive oxidation of aromatic structures to weaken the van der Waals interaction between the graphene sheets for their exfoliation into single layers and dispersion in solutions [13] which can be further aided by sonication [52]. However, these oxidation procedures generate toxic gases such as nitrogen dioxide, dinitrogen tetroxide, or chlorine dioxide, the latter being explosive [2].

Recently, GO has also been synthesized using the “bottom-up” method with strong oxidizers. This process is safer, simpler, and more environmentally friendly than the “top-down” method [53]. For instance, Tang-Lau et al. [53] used glucose as the sole reagent and the bottom-up assembly technique to grow GO. Moreover, this method has an important advantage because the layer thickness can be controlled by adjusting the growth parameters. An EC alternative was explored by Pei et al. [54] using electrolytic oxidation by dipping graphite paper in H_2_SO_4_ for EC intercalation, followed by exfoliation to obtain GO, which was also conducted via electrolysis. Excess H_2_SO_4_ can be fully recycled, thereby presenting an environmentally friendly, efficient, and low-cost method of GO production.

**Fig. 2:**
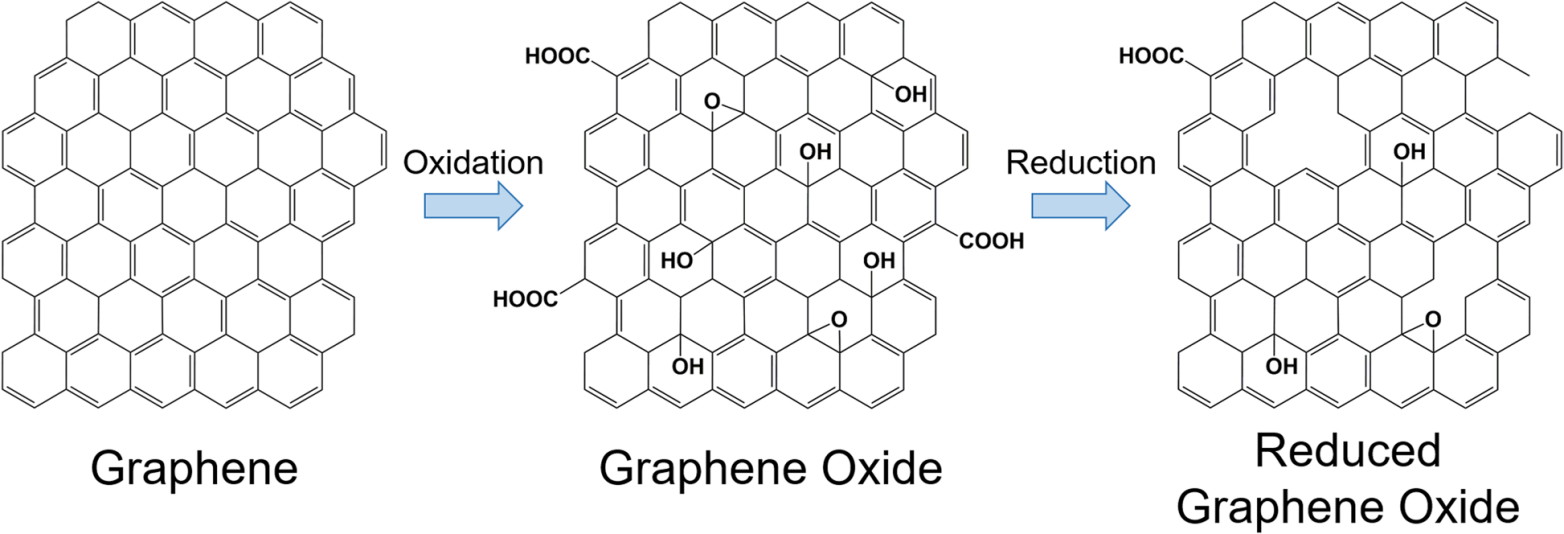
Chemical structures of graphene, graphene oxide, and reduced graphene oxide and their synthetic processes

Furthermore, GO can be reduced to acquire rGO. Reduction eliminates the majority of the carbonyl, carboxyl, hydroxyl, and epoxy groups on the GO sheets, as illustrated in Fig. 2 [55–57]. However, the reduction process cannot produce pristine graphene because of the presence of residual oxygen-functional groups and defects [58]. Moreover, rGO can be prepared using various methods. The most popular method is chemical reduction, although other methods are also available, including thermal reduction, electrochemical reduction, and photothermal reduction [28, 59–64]. The partial reduction of GO can allow the tuning of rGO properties, such as molecular adsorption [65], electrical conductivity [66, 67], and light adsorption [68], as needed.

Chemical reduction is the most popular method for the production of GO-/rGO-based nanocarriers, as it is relatively fast and easy [62, 69]. Traditionally, the chemical reduction to prepare rGO involves hydrazine hydrate, which is highly effective. However, because of their high toxicity, many alternatives have been explored, including acids, alkalis, oxygen-containing reducing agents, amino acids, and microorganisms [60, 70]. Generally, the reduction requires high temperature (maximum 100 °C), although the reaction time varies depending on the chosen reagent [60, 70]. The type of reducing agent critically influences the reduction degree and properties of the prepared rGO [71].

### Structure

Dékány’s model is a well-recognized structure for GO comprising two domains, including trans-linked cyclohexyl species interspersed with tertiary alcohols and 1,3-ethers, alongside a corrugated network of keto/quinoidal species [1, 49]. The model suggests that the corrugating nature of the carbon network is interrupted by the trans-linked cyclohexyl regions and functionalized by tertiary alcohols and 1,3-ethers [49]. Different models of GO illustrate the variations in the degree of oxidation, structures, and properties depending on the starting materials (graphite source) and oxidation protocol [49]. Furthermore, all the GO structural models contain oxygen groups at the edges of the graphene sheets and above and below the basal plane [49, 72].

Moreover, rGO remains structurally similar to GO, with only the elimination of most oxygen-containing functional groups and an increase in the percentage of sp^2^ hybridization being the main differences [57]. The elimination of oxygen-containing functional groups creates vacancies in the GO sheet structure, which is evident from the increase in the ratio of the D to G peak intensity in the Raman spectrum [57, 73]. Second-order Raman scattering is represented by the 2D band where its intensity, width, and position relates to the stacking of GO and rGO sheets [74, 75]. Finally, sp^3^-hybridisation is dependent on the relative intensity of the D band compared to that of the G band [76]. It should be noted that rGO is less susceptible to photodegradation than GO because it contains fewer oxygen-containing functional groups [72].

## Properties of graphene oxide and reduced graphene oxide

### Physical properties

Initially, GO attracted interest in the nanocarrier field because of its good colloidal stability and large surface area. The 2D structure of GO lends itself to a large surface area, which results in a high loading capacity, which is a property shared by rGO [77]. However, unlike GO, rGO exhibits poor colloidal stability and readily aggregates within a few hours of dispersion in water [78]. The percentage of C-O and C = O bonds in rGO affects its colloidal stability. The better hydrophilicity of GO is attributed to the presence of abundant oxygen-containing functional groups in its structure compared to that of rGO [78, 79]. Nevertheless, rGO with improved colloidal stability can be produced depending on the reducing agents and resulting surface properties [80]. Additionally, graphene-derived materials are known to have high mechanical strength and flexibility; monolayer GO and rGO have an effective elastic modulus of approximately 207.6 [81] and 250 GPa [82] respectively. Finally, rGO was shown to have more thermal stability due to its comparatively less deoxygenated state [83].

### Electrical properties

With the possibility of counteracting its colloidal instability, rGO has attracted interest in the nanocarrier field owing to its high electrical conductivity. In addition, GO is considered an insulator because of its large defects in sp^2^ carbon bonds, whereas rGO can display high electrical conductance resembling that of pristine graphene [79]. The change from an insulator to a highly conductive material has been ascribed to the reduction in oxygen functional groups and the high percentage of sp^2^ hybridization [68]. An increase in the C/O ratio increased the conductivity, allowing the rGO conductivity to be tuned [66]. Furthermore, GO displays a negative differential resistance with varying results depending on the relative humidity, air pressure, and applied voltage [84].

### Optical properties

Both GO and rGO benefit from the absorbance of visible and ultraviolet light, with an observed emission wavelength in the range of 350–650 nm [85]. The absorbance peaks of GO and rGO are approximately 230 [85, 86] and 260 nm, respectively [87, 88]; however, both have a wide absorbance in the range of 200–900 nm [85, 87, 88]. Depending on the excitation wavelength, a range of fluorescent emissions can be achieved [89]. Furthermore, the GO and rGO emission peaks can be further tuned based on the number and type of attached functional groups [89, 90].

### Photothermal properties

Both GO and rGO effectively absorb near-infrared (NIR) light, which is a biocompatible light source that penetrates tissues. Moreover, GO and rGO convert the absorbed NIR light energy to heat, increasing the temperature in GO and rGO and their surrounding media [85, 88, 91, 92]. While both GO and rGO can absorb NIR, rGO is more effective [91] likely because of the red shift in the absorbance peak from approximately 230 to 260 nm [87, 88].

### Biocompatibility

Opinions on the cytotoxicity and biocompatibility of GO are contradictory because of the varying effects depending on the concentration used; specifically, GO is cytotoxic at higher concentrations. However, GO generally has low cytotoxicity at concentrations below 4 μg/mL [93, 94]. Moreover, rGO is less cytotoxic than GO even at higher concentrations [94, 95]. This cytotoxicity could be attributed to membrane damage caused by the sharp edges of the nanoparticles and induced oxidative stress [96]. Research has indicated that cytotoxicity of GO is also dependent on the particle size and level of aggregation [97]. Meanwhile, high carbon radical density has been associated with the increased toxicity of GO via lipid peroxidation and membrane damage [98]. Therefore, the level of cytotoxicity can be controlled by tuning all these factors. Genotoxicity of GO/rGO nanoparticles is also a concern, with research indicating that both direct and indirect mechanisms exist in DNA damage [99]. Although the surface functionalization of GO affects its eventual clearance, GO particles aggregate in organs, potentially causing structural damage [100, 101]. Induced by GO, platelet aggregation causing thromboembolism is also a concern, although rGO causes significantly less platelet aggregation [102]. In vivo studies in mice [103] and fish [104] resulted in toxic effects, demonstrating that further studies on GO/rGO biocompatibility are needed. Notably, GO/rGO could stimulate the immune response by inducing cellular activation and cytokine production [105].

In addition, both GO and rGO can displayed antibacterial properties that may be attributed to the previously mentioned membrane damage and oxidative stress as the particles can aggregate on bacterial cells. The degree of such antibacterial effects depends on the oxidative capacity, size [96], concentration, and contact time of the GO or rGO particles with the bacteria [106]. A comparison between the cytotoxicity of GO/rGO sheets against bacterial and mammalian cells has been performed, proving that they are more cytotoxic to bacteria at similar concentrations [107]. However, the relative size ratios between the sheets and cells used in the study were not mentioned. Another study showed that a positive zeta potential of approximately 20 ± 2 mV was particularly effective in capturing gram-negative pathogens, such as E. coli, while being ineffective for gram-positive pathogens, such as S. Aureus [43]. Notably, research regarding the antibacterial properties of GO/rGO generally uses significantly higher concentrations than the 4 μg/mL, which is regarded as the maximum non-cytotoxic concentration [43, 108].

Both GO and rGO have strong interactions with single-stranded DNA (ssDNA) through hydrophobic and π-π stacking interactions [109–111]. However, functionalization with positively charged molecules is necessary for interactions with double-stranded DNA (dsDNA) to allow electrostatic interactions [112–114]. Finally, the biodegradation of GO occurs under both aqueous [115, 116] and enzymatic conditions [117–120]. Enzymatic conditions, including eosinophil peroxidase [117], myeloperoxidase [119], and lignin peroxidase [120] accelerate the process through enzymatic digestion. The effects of GO biodegradation can be observed within hours of exposure to enzymes [117, 119, 120]. Additionally, rGO is affected by enzymatic degradation, although at a slower rate, which might be due to its reduced level of oxidization [120]. Research indicates that GO degradation is mediated by neutrophils and macrophages, and that the resulting degradation products are neither cytotoxic nor genotoxic [119].

## Functionalizing graphene oxide and reduced graphene oxide

Solubility, biocompatibility, drug-loading capacity, and release efficiency are considered to enhance the functionality and reduce toxicity of graphene-based nanocarriers [121, 122]. Recently, the surface functionalization of GO and rGO has been studied to improve their biological properties and enhance their potential efficiency for therapeutic use [123]. There are two main approaches for modifying the GO or rGO surfaces. First, covalent functionalization is typically carried out using chemical reactions with carboxylic, epoxy, and hydroxyl groups present on the GO surfaces using various coupling agents [124]. Second, noncovalent functionalization is usually carried out with inorganic nanoparticles and other molecules, such as polymers, drugs, proteins, and small molecules, on the GO or rGO surface through hydrophobic, van der Waals, electrostatic, and H-bonding interactions [121].

### Covalent functionalization

Covalent functionalization is an approach for grafting polymers or immobilizing biomolecules onto GO sheets, based on different chemically reactive functionalities on the basal plane (epoxy and hydroxyl) and sheet edges (carboxylic acid) [123, 125]. The surface modifications with stable covalent bonds improve the stability of immobilized proteins, enzymes, drugs, or small molecules in the system to improve GO properties, such as biocompatibility and loading stability [126]. A few studies have been conducted on the biocompatibility of functionalized GO for the delivery of a series of drugs, including synthetic compounds, proteins, antibodies, and genes, through covalent functionalization. In recent years, the application of GO as a carrier for small interfering RNA (siRNAs) has demonstrated great potential.

**Fig. 3:**
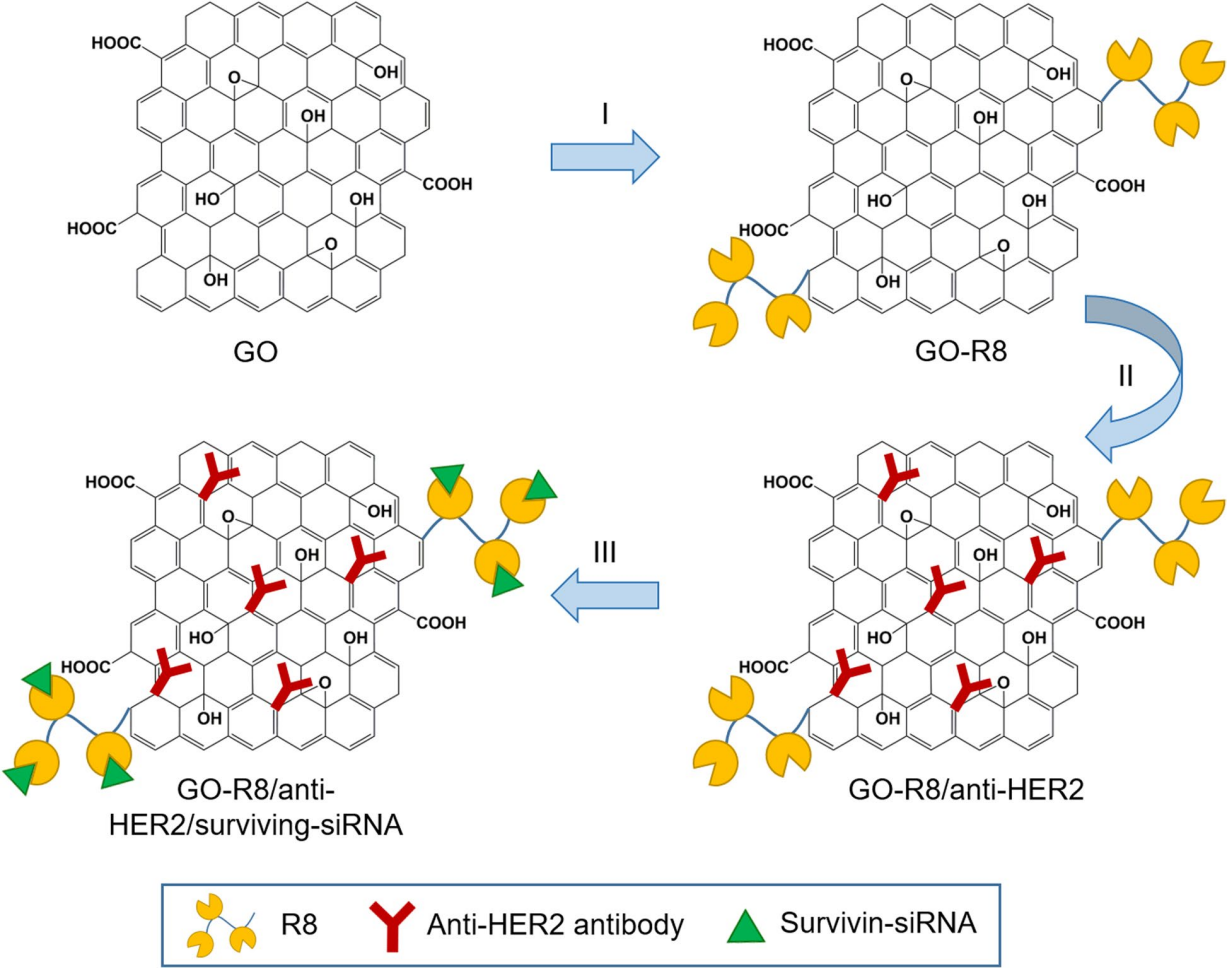
Schematic illustration of graphene oxide (GO) functionalization with octaarginine (R8) and anti-HER2 antibody. Notes: Adapted from Wang X, Sun Q, Cui C, Li J, Wang Y. Anti-HER2 functionalized graphene oxide as a survivin-siRNA delivery carrier inhibits breast carcinoma growth in vitro and in vivo. Drug Des Devel Ther. 2018;12:2841–2855 [127]

Wang et al. [127] prepared octaarginine (R8) and anti-HER2 antibody-functionalized GO using covalent conjugation (Fig. 3) as a novel gene delivery system for tumor therapy. In addition, R8 was modified onto GO surfaces as a cell-penetrating peptide to enhance the effect of siRNA delivery, whereas anti-HER2 was labeled together to bind with HER2. Furthermore, GO-R8/anti-HER2/survivin-siRNA is a potentially efficient gene-silencing carrier for siRNA delivery in cancer therapy in vitro and in vivo.

In a study by Li et al. [128] a novel nanogene delivery system into HeLa cells was prepared by functionalizing GO with R8 and cRGDfV peptides, which could increase the stability, electropositivity, transfection efficiency, cytocompatibility, and tumor inhibition [128]. In addition, Jana et al. [123] successfully achieved dual covalent chemical functionalization of GO with tris-[nitrilotris(acetic acid)] and biotin. This functionalized GO served as a carrier for cellular delivery of oligohistidine- and biotin-tagged biomolecules such as proteins.

Functionalization of GO with polymers can improve the drug release efficiency at tumor sites when the modified carriers reach the target cells, resulting in more effective therapy. For example, Gao et al. [129] developed a GO-modified polysebacic anhydride (GO/PSA) composite as a drug carrier to improve controlled release properties. GO/PSA composites were synthesized via Steglich esterification, which occurred between PSA and the suspended hydroxyls in GO to yield esters. The GO to PSA ratio affected the drug release duration, and the composites at the optimal ratio exhibited a long-term release of up to 80 days. The effective drug release rate exceeded 95%.

**Fig. 4:**
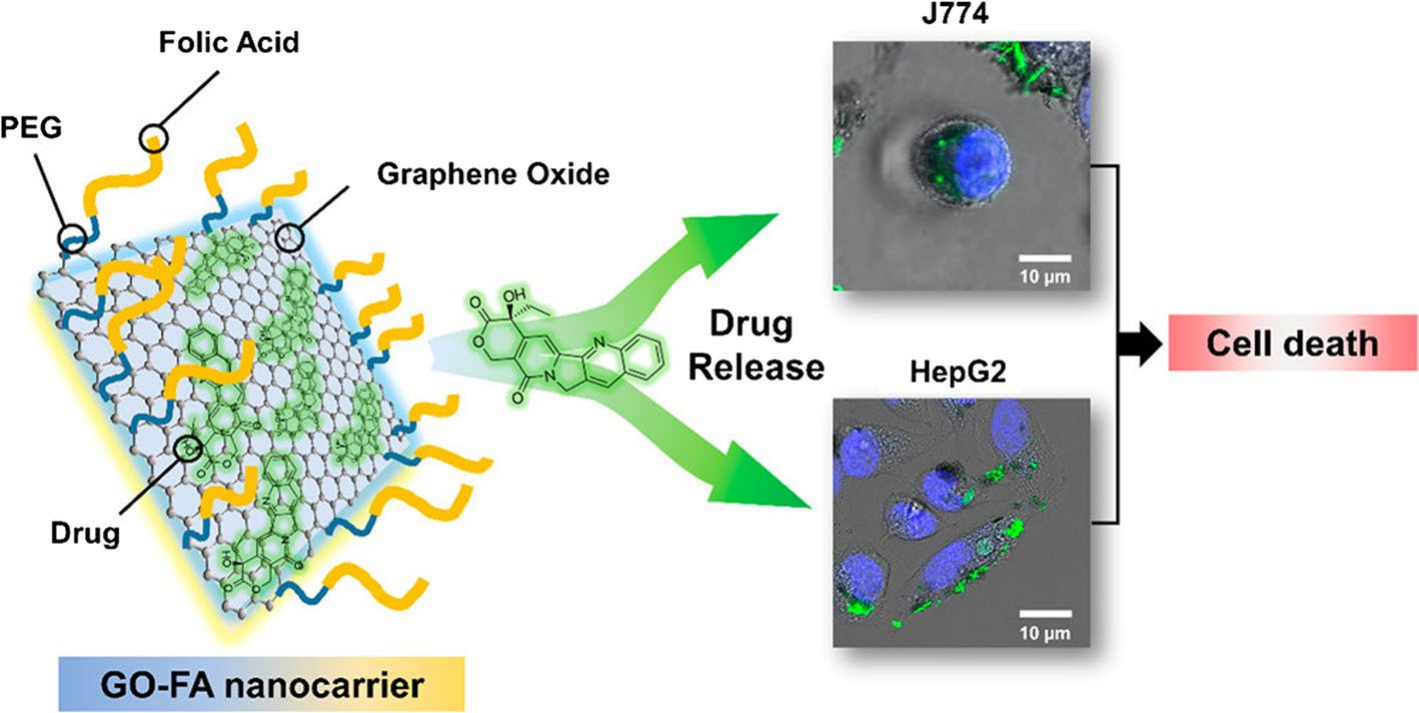
Schematic of graphene oxide (GO) functionalization with folic acid (FA)-linked polyethylene glycol (PEG) and preclinical screening tests for two tumor cell models. Notes: Reprinted with permission from de Sousa, Visani de Luna, Fonseca, Giorgio, and Alves. Folic-acid-functionalized graphene oxide nanocarriers: Synthetic approaches, characterization, drug delivery study, and antitumor screening. ACS Appl Nano Mater. 2018;1(2):922–932. [122] Copyright 2018, American Chemical Society

Similarly, de Sousa et al. [122] produced nanocarriers consisting of GO functionalized with folic acid (FA) for drug delivery (Fig. 4). In this system, FA was linked to polyethylene glycol (PEG) and coupled to the GO surface. The dynamic release of drugs from the nanocarrier was examined under two physiological conditions using sink conditions and camptothecin (CPT) as a model drug. Toxicity screening of the nanocarrier was performed in vitro for two tumor cell models that promoted tumor cell death by apoptosis.

**Fig. 5:**
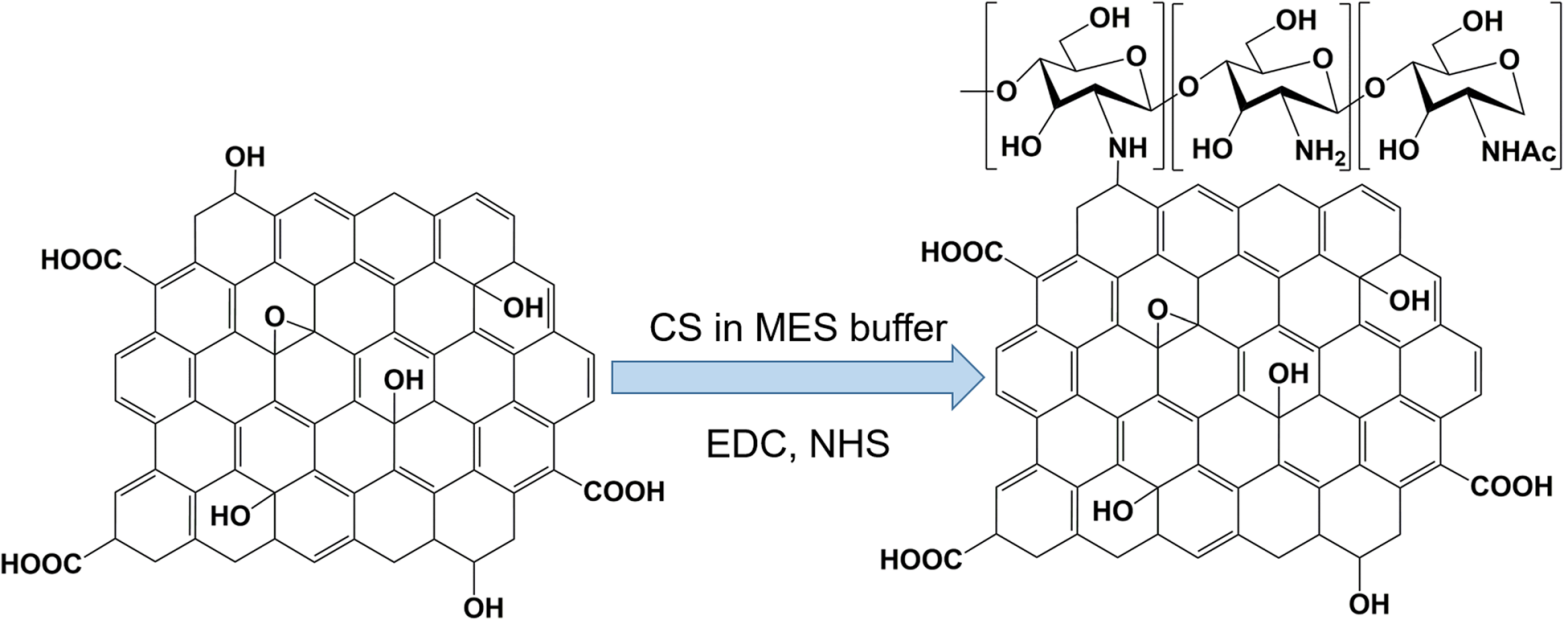
Synthesis of chitosan (CS)-functionalized graphene oxide. MES buffer is 2-(N-morpholino)ethanesulfonic acid buffer, EDC is 1-Ethyl-3-(3-dimethylaminopropyl)carbodiimide, and NHS is N-Hydroxysuccinimide. Note: Adapted from Bao H, Pan Y, Ping Y, et al. Chitosan-functionalized Graphene Oxide as a nanocarrier for drug and gene delivery. Small. 2011;7(11):1569–1578 [126]

Bao et al. [126] reported the use of a facile amidation process to synthesize the GO covalently functionalized with chitosan (CS) for drug and gene delivery (Fig. 5). Grafting CS onto GO sheets improves the solubility and biocompatibility of GO. Moreover, inorganic nanoparticles, such as iron oxide, have been conjugated to the GO surface to enhance *T*_*2*_-weighted magnetic resonance (MR) imaging contrasts.

**Fig. 6:**
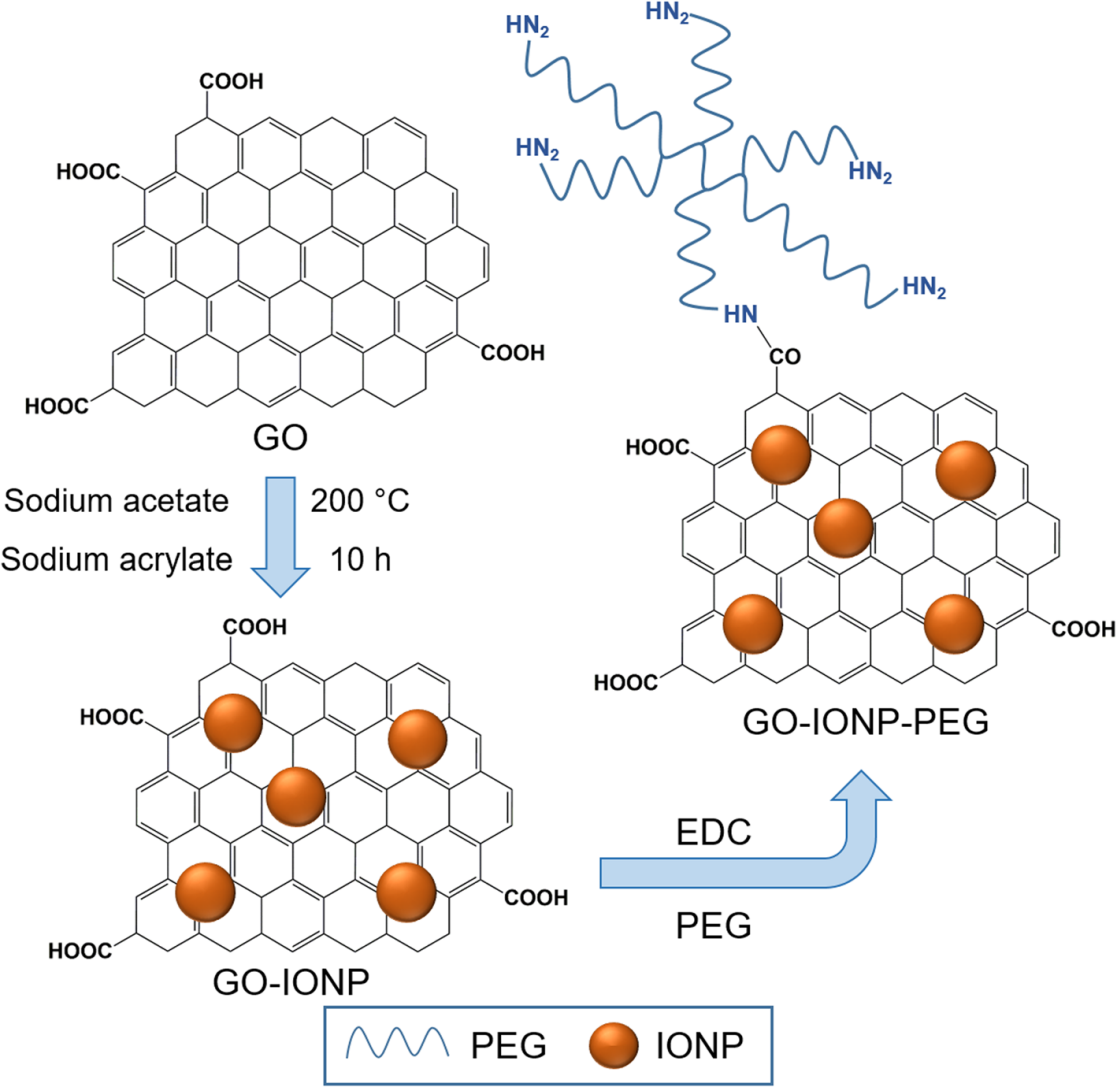
Schematic illustration of synthesis of graphene oxide (GO) and iron oxide (IONP) nanocomposite functionalized with polyethylene glycol (PEG). Notes: Adapted from Ma X, Tao H, Yang K et al. Functionalized graphene oxide-iron oxide nanocomposites for magnetically targeted drug delivery, photothermal therapy, and magnetic resonance imaging. Nano Res. 2012;5(3):199–212 [130]

Ma et al. [130] reported a multifunctional superparamagnetic GO-iron oxide hybrid nanocomposite (IONP) that was further functionalized with biocompatible PEG, which displayed increased drug loading capacity and strong *T*_*2*_-weighted MR contrast in a mouse tumor and liver. Specifically, GO-IONP-PEG was synthesized by the chemical deposition of IONPs onto GO sheets and the subsequent functionalization of GO with branched PEG through amide bonds, as illustrated in Fig. 6. However, covalent functionalization is not popular for immobilizing biomolecules onto rGO surfaces because of the lack of oxygen-containing functional groups on the surface of rGO.

### Noncovalent functionalization

In general, the noncovalent functionalization of GO and rGO involves van der Waals forces, π-π interactions, hydrogen bonding, and electrostatic interactions with polymers or biomolecules [131]. Noncovalent interaction is a simple approach for functionalization with various molecules without impairing the internal structure and affecting important properties, such as electrical conductivity and mechanical strength, of GO or rGO after functionalization with other materials [132].

**Fig. 7:**
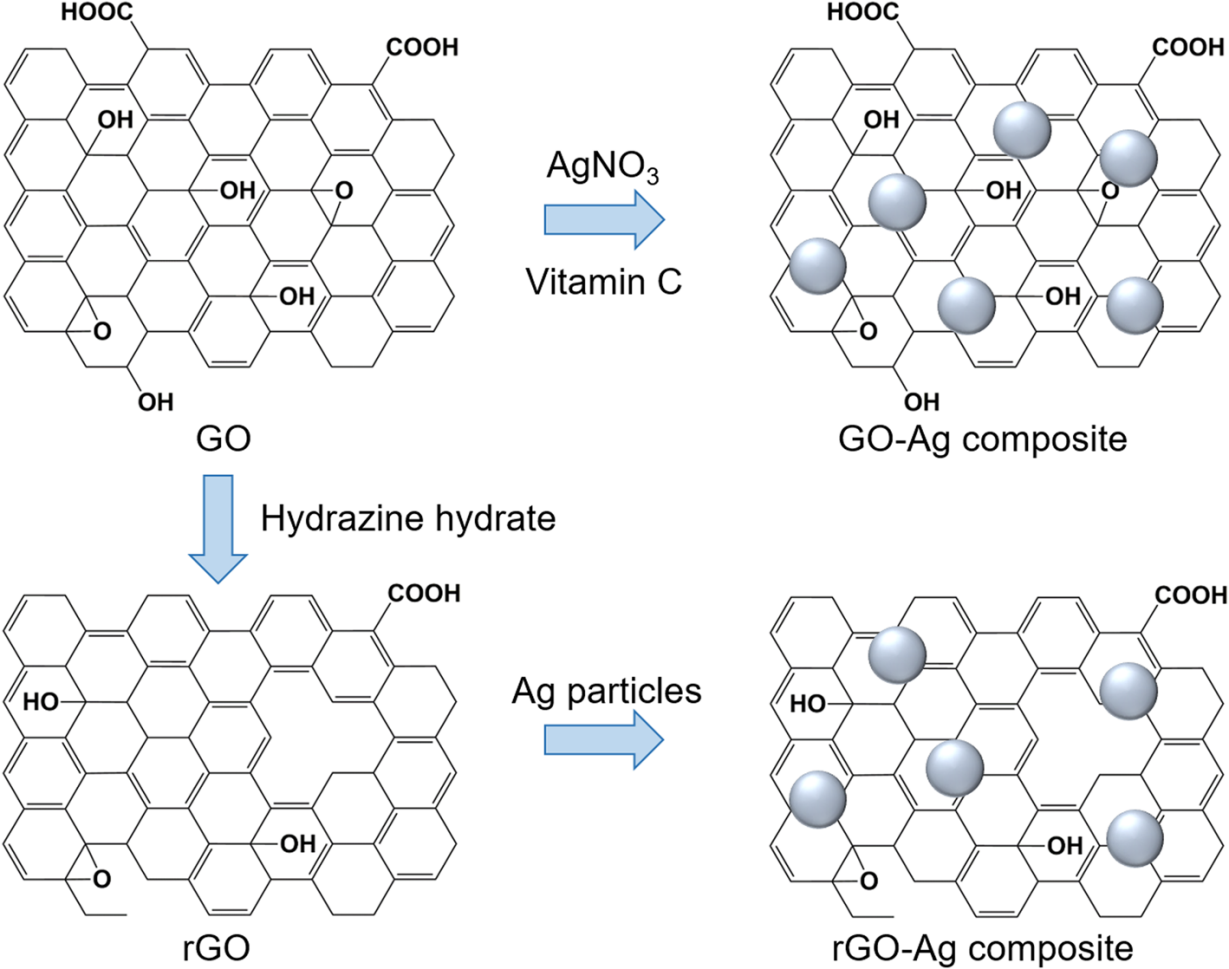
Schematic illustration of synthesis of graphene oxide (GO)/reduced graphene oxide (rGO) and silver (Ag) composite. Notes: Adapted from Kavinkumar T, Varunkumar K, Ravikumar V, Manivannan S. Anticancer activity of graphene oxide-reduced graphene oxide-silver nanoparticle composites. J Colloid Interface Sci. 2017;505:1125–1133 [133]

An example of functionalization with inorganic nanoparticles as carriers for anticancer applications in the form of silver (Ag) nanoparticles was reported by Kavinkumar et al. [133]. The GO/rGO-Ag nanoparticle composites were obtained by a chemical route using vitamin C as the reducing agent (Fig. 7), demonstrating significant cytotoxicity toward A549 cells. Therefore, this approach has been suggested for cancer prevention and treatment. Usually, noncovalent functionalization of the GO surface with polymers, biomolecules, and drugs can be achieved by either wrapping or absorption, mostly via π-π interactions. The most popular biocompatible polymer used to modify the GO surface is PEG, as it can be easily connected with various anticancer drugs and has continuous release behaviors.

Kazempour et al. [124] studied the release profile of doxorubicin (DOX) at two different pH levels from a biocompatible carrier of PEG-functionalized GO (GO-PEG). They found that the GO-PEG hybrid exhibited high drug loading and more release at acidic pH (5.8) because of two kinds of possible H-bonding between the drug and carrier, whereas at neutral pH (7.4), four kinds of H-bonding existed between the drug and carrier; hence, negligible release occurred.

**Fig. 8:**
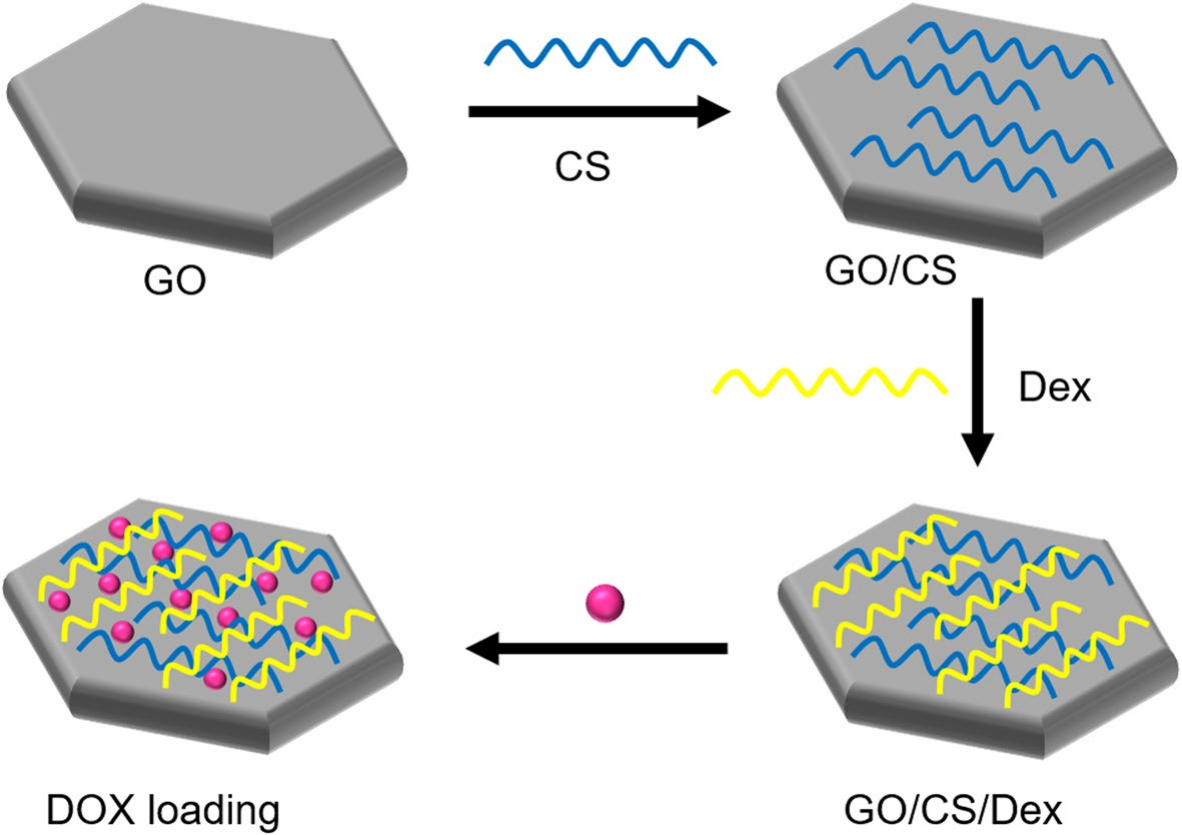
Schematic illustration of synthesis of graphene oxide (GO) nanosheets functionalized with chitosan (CS) and dextran (Dex) and loaded with doxorubicin hydrochloride (DOX). Notes: Adapted from Xie M, Lei H, Zhang Y, et al. Noncovalent modification of graphene oxide nanocomposites with chitosan/dextran and its application in drug delivery. RSC Adv. 2016;6(11):9328–9337 [134]

Several studies have reported a new class of GO-based carriers that use a layer-by-layer (LbL) technique involving the alternate deposition of oppositely charged polyelectrolytes on GO sheets via electrostatic interactions for surface functionalization. For example, Xie et al. [134] chose two natural linear polymers (positively charged CS and negatively charged dextran) as oppositely charged polyelectrolytes to prepare polyelectrolyte-stabilized GO nanocomposites for drug delivery (Fig. 8). Li et al. [135] used an LbL assembly to synthesize GO nanoassemblies with different types of polyelectrolytes, including poly-L-lysine (PLL), polystyrene sulfonate, PLL-PEG, poly(lactic-co-glycolic acid)-PEG, and DNA oligonucleotides.

Drugs and other biomolecules can be functionalized onto GO and rGO surfaces via noncovalent conjugation. Functionalization of GO nanocolloids with bovine serum albumin protein was reported by Sima [121] for antitumor drug delivery to melanoma cells. This type of functional bioplatform presents high potential as a miniaturized high-throughput platform for drug screening and testing cancer cell responses to different drugs and drug doses in precision medicine applications. Tan et al. [125] synthesized immobilized glutaryl-7-aminocephalosporanic acid acylase onto GO as a carrier to enhance the stability of the immobilized enzyme as a catalyst. Mu et al. [136] elucidated cellular uptake mechanisms by investigating the cellular uptake of protein-coated GO nanosheets. These findings provide fundamental information that sheet-shaped GO nanostructures with protein coatings can adhere to cell surfaces and undergo size-dependent internalization, facilitating nanomedicine and nanotoxicity studies.

**Fig. 9:**
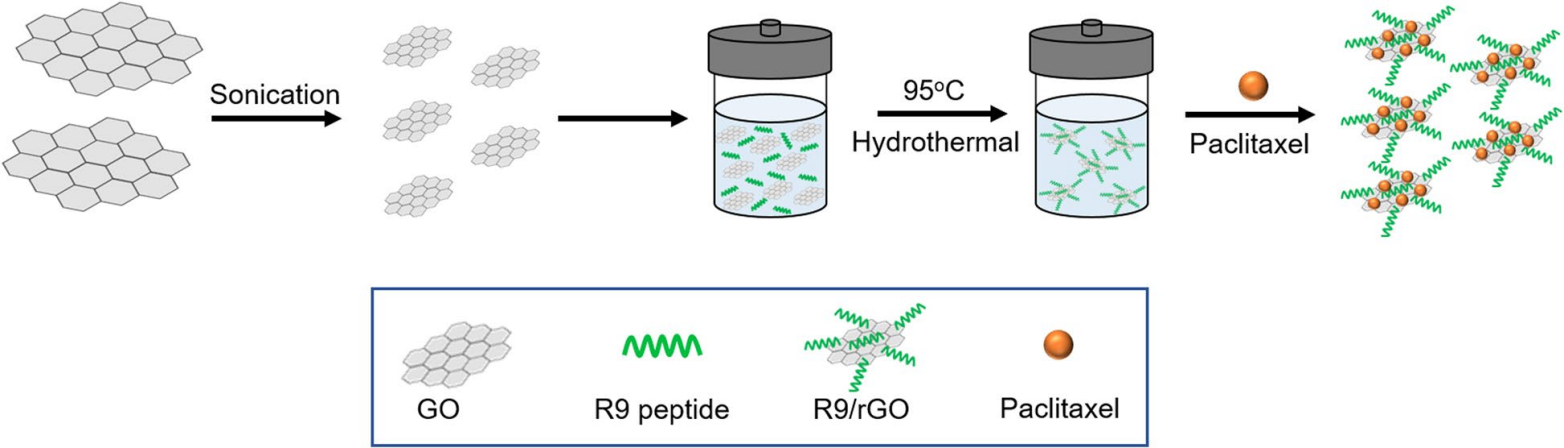
Schematic illustration of reduced graphene oxide (rGO)-based nanocarrier grafted with R9 peptide and loaded with Paclitaxel. Notes: Adapted from: Hashemi M, Yadegari A, Yazdanpanah G, Jabbehdari S, Omidi M, Tayebi L. Functionalized R9–reduced graphene oxide as an efficient nano-carrier for hydrophobic drug delivery. RSC Adv. 2016;6(78):74,072–74,084 [137]

A challenging issue for loading hydrophobic drugs onto graphene-based nanocarriers has been addressed for advanced drug delivery systems. Hashemi et al. [137] suggested paclitaxel (PAC) drug loading on R9 peptide-rGO through hydrophobic interactions (Fig. 9) as a green and simple method of achieving an applicable graphene-based drug delivery system to improve the transportation of hydrophobic anticancer drugs. Moreover, a few studies have reported conjugation of DOX, as a model drug, on the rGO surface via strong π-π stacking interactions [138, 139] in drug delivery systems. In addition to DOX, different chemotherapeutic drugs, such as CPT [140, 141], PAC [137, 142], and mitoxantrone [143] could be loaded onto rGO by hydrophobic and π-π stacking interactions to inhibit the growth rates of cancer cells.

## Applications of graphene oxide- and reduced graphene oxide-based composites

**Fig. 10:**
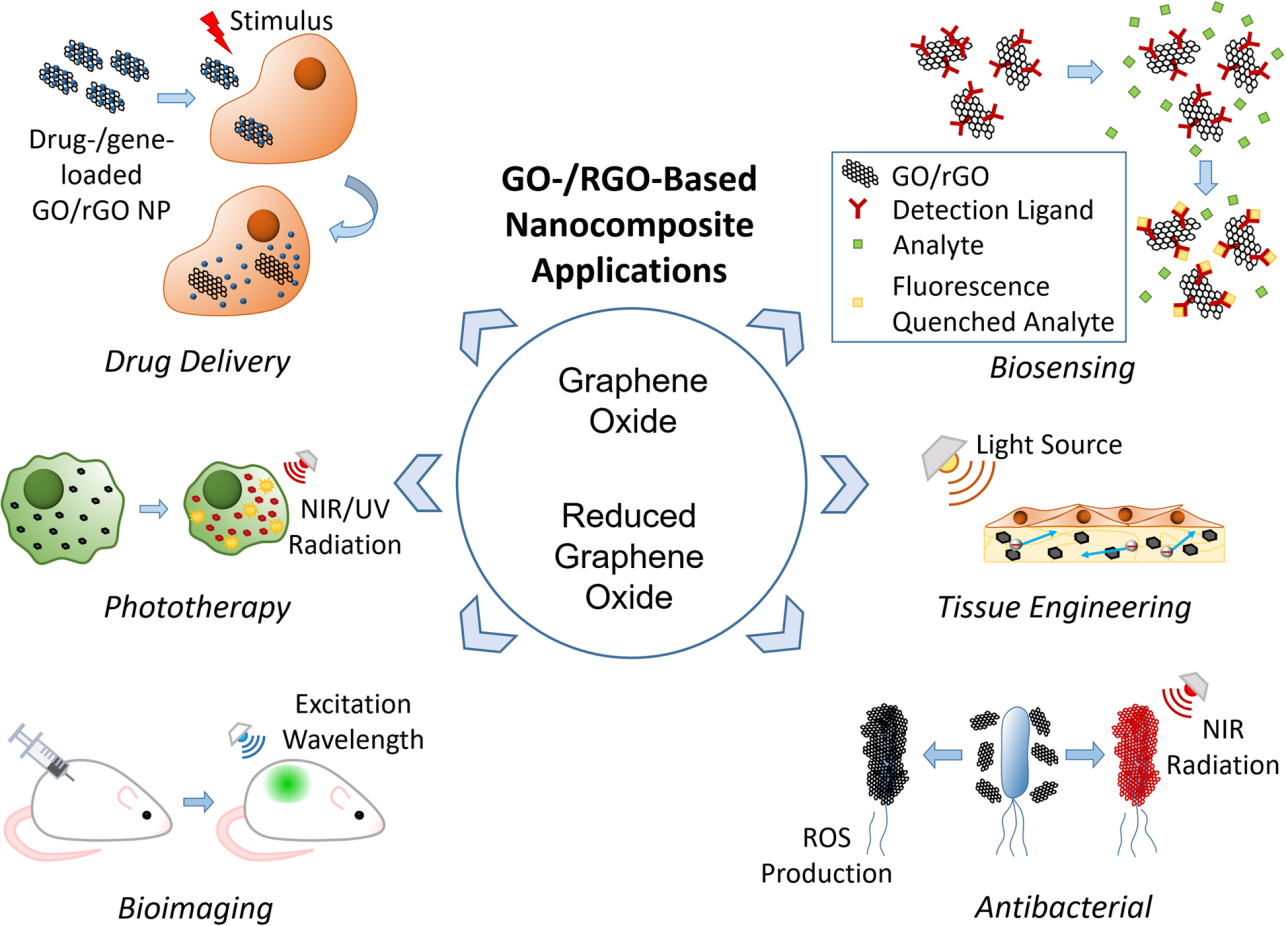
Schematic illustrations of biomedical applications of graphene oxide-/reduce graphene oxide-based nanocarriers

The biomedical applications of GO-/rGO-based composites can be classified into six groups: drug and gene delivery, phototherapy, bioimaging, biosensing, tissue engineering, and antibacterial applications. Each application uses one or several properties of GO and rGO as presented in Fig. 10, whereas some properties are useful for multiple applications. Thus, GO- and rGO-based nanocarriers have high potential to function as multipurpose carriers that can be applied in any combination of the listed applications. This function is particularly attractive because it can reduce the number of steps required in diagnosis/treatment, creating a more efficient and streamlined process.

### Drug and gene delivery

As described earlier, GO has been explored as a drug and gene carrier owing to its colloidal stability, relatively low toxicity, large surface area, and high loading stability. As rGO shares the same traits with GO except for its colloidal stability, it has also been explored as a carrier; however, its instability issue can be easily rectified with correct functionalization [114, 144]. Additionally, functionalization can be used to improve carrier cell targeting abilities [33, 145, 146]. Furthermore, both GO and rGO nanocarriers are pH-responsive [33, 145, 147, 148] and photo-responsive [114, 141, 146, 149], allowing for controlled/smart drug release. Drug and gene delivery systems using GO/rGO and their properties are listed in Table 1.

**Table 1:** List of graphene oxide and reduced graphene oxide composites and their drug and gene delivery applications

Composite	Drug Type	Delivery Stimulus	Target Cell	Applications	Study Type	Additional Functions	Ref
**rGO-MPAH-FA**	pDNA	NIR	HEK-293A	Gene therapy	In vitro	-	[114]
**BPBA@GA-rGO**	GA	pH	A549	Chemotherapeutic	In vitro	-	[150]
**rGO/KGN@Ge**	KGN	-	ADSC	Repairing cartilage defect	In vitro	-	[151]
**rGO/CS**	5-FU CUR	-	HT-29	Chemotherapy	In vitro	-	[152]
**rGO@MSN**	DOX	pH	A549	Cancer therapy	In vitro	Phototherapy	[153]
**rGO/β-carotene**	Nrf2	-	Hepatic stellate cells	Ameliorate hepatic fibrosis and influences Nrf2 signaling	In vivo	-	[154]
**MrGO-AA-g-4-HC**	CPT 4-HC	pH	MCF7 WS1	Chemo-photodynamic therapy Cancer therapy	In vitro/ In vivo	Phototherapy	[141]
**PEG-BPEI-rGO**	DOX	NIR	PC-3	Cancer treatment	In vitro	Phototherapy	[149]
**CHA-rGO**	DOX	-	KB epidermal carcinoma	Cancer treatment	In vitro/ In vivo	-	[144]
**rGO/β-CD**	Azo-C_60_	UV	PC-12	Protection cytotoxicity from nitric oxide	In vitro	-	[155]
**rGO/MSN/PDA**	DOX	pH NIR	MHCC97-L MHCC97-H	Chemo-photothermal therapy	In vitro	Phototherapy	[156]
**rGO-PDA**	Ara	NIR	HeLa	Antitumor therapy	In vitro/ In vivo	Phototherapy	[157]
**rGO-PLPEG**	siRNA	-	MCF7	Gene therapy	In vitro	-	[158]
**CUR@HSA-MNPs@rGO**	DOX	pH	SH-SY5Y	Cancer treatment	In vitro	-	[145]
**rGO/HA-SP**	DOX	-	MDCK A549	Cellular imaging Cancer treatment	In vitro/ In vivo	Bioimaging	[33]
**PEG-rGO**	ssRNA	-	HeLa	Gene therapy	In vitro	-	[17]
**Zn-dopamine-rGO**	DOX	pH	T-47D MCF10A	Cancer treatment Antibacterial	In vitro	Antibacterial	[159]
**Gd-rGO**	5-FU	-	H1299	Optical coherence tomography Magnetic resonance imaging	In vitro	Bioimaging	[160]
**(CA-BODIPY)-PPDN/rGO**	DOX	pH Thermal	MDCK MDA-MB-231	Cellular imaging Cancer treatment	In vitro	Bioimaging	[161]
**CuS(DOX)-GO-HA**	DOX	pH NIR	SCC-7 MDA-MB-231 BT-474	Cancer therapy	In vitro/ In vivo	Phototherapy	[146]
**GO-PEG**	DOX	pH	CAL-27 SCC-25 HOK	Cancer therapy	In vitro	-	[147]
**GO@Ge**	PAC	pH	L929 MCF7	Chemo-photothermal therapy	In vitro	Phototherapy	[148]
**MGO-PEG-CET**	DOX	pH	CT26	Chemo-phototherapy	In vitro/ In vivo	Phototherapy	[162]
**GO/Red blood cell membrane**	DOX	pH	MCF7	Cancer chemotherapy	In vitro/ In vivo	-	[163]
**GO/Fe**_**3**_**O**_**4**_	MTX	-	Caov-4 HeLa MCF7	Cancer chemotherapy	In vitro	-	[164]
**GO/Au-PEG-PLA**	miR-101	NIR	MCF7 MDA HU02	Gene therapy	In vitro	Phototherapy	[165]
**GO-PEG-PLA**	miR-101	NIR	MCF7 MCF10A MDA-MB-231 HU02	Chemo-photothermal therapy	In vitro	Phototherapy	[166]

*Abbreviations: 4-HC* 4-hydroxy coumarin, *5-FU* 5-Fluorouracil, *AA* Allylamine, *Ara* Cytarabine hydrochloride, *Au* Gold, *Azo-C60* Azobenzene-functionalized fullerene, *BODIPY* Boron-dipyrromethene, *BPBA* Biotin-adorned poly-(ethylene oxide)bis-(amine), *BPEI* Branched polyethylenimine, *CA* Catechol, *CD* Cyclodextrin, *CET* Cetuximab, *CHA* Cholesteryl hyaluronic acid, *CPT* Camptothecin, *CS* Chitosan, *CUR* Curcumin, *CuS* Copper sulfide, *DOX* Doxorubicin, *FA* Folic acid, *Fe*_*3*_*O*_*4*_ Iron oxide, *GA* Gallic acid, *Gd* Gadolinium, *Ge* Gelatin, *GO* Graphene oxide, *HA* Hyaluronic acid, *HC* Hydroxycoumarin, *HSA* Human serum albumin, *KGN* Kartogenin, *MGO* Magnetic graphene oxide, *MNPs* Magnetic nanoparticles, *MPAH* Modified poly(allylamine hydrochloride), *MrGO* Magnetic reduced graphene oxide, *MSN* Mesoporous silica nanoparticle, *MTX* Methotrexate, *NIR* Near-infrared, *Nrf2* Nuclear factor erythroid 2-related factor-2, *PAC* Paclitaxel, *PAH* Polyallylamine hydrochloride, *PDA* Polydopamine, *pDNA* Plasmid DNA, *PEG* Polyethylene glycol, *PET* Positron emission tomography, *PLA* Poly-L-arginine, *PLPEG* Phospholipid-PEG, *PPDN* PEG–g–poly (DMA–co–NIPAAm), *R8* Octaarginine, *rGO* Reduced graphene oxide, *SP* Spiropyran, *Zn* Zinc

Vinothini et al. [141] created an rGO nanocarrier decorated with magnetic nanoparticles and CPT, which was also cross-linked with 4-hydroxycoumarin (HC) using allylamine to explore the rate of release of CPT and 4-HC under various pH conditions. The rate of release increased with lowering of pH. Liu et al. [153] created a DOX-loaded mesoporous silica-coated rGO composite to release DOX under acidic conditions of pH 5.0 with the addition of NIR irradiation at 808 nm. NIR irradiation significantly increased the release rate of DOX, resulting in a highly effective nanocarrier for controlled drug release. For gene delivery, Assali et al. [166] designed a cationic GO-based nanocarrier which carried miRNA-101 which suppressed Stathmin1 protein in cancer cells, thereby inducing apoptosis and downregulating autophagy. Furthermore, the particles were covalently decorated with PEG and poly-L-arginine to increase internalization and cause reduction at the surface of the GO nanocarriers, finally increasing their NIR absorption, and making them suitable for phototherapy.

### Phototherapy

Phototherapy generally involves two forms: photodynamic and photothermal therapy, both of which use the light-absorbent properties of GO and rGO. Photodynamic therapy relies on a light source to induce singlet oxygen radical generation [141, 167], and photothermal therapy relies on NIR as an energy source for heat emission [153, 168]. Among the examples listed in Table 2, a clear preference for using rGO for photothermal therapy exists, likely because of its higher NIR absorbance, making it more effective in treatment. Phototherapy is likely to be used in conjunction with other therapies, specifically drug delivery, for effective cancer treatment.

**Table 2:** List of graphene oxide and reduced graphene oxide composites and their phototherapy applications

Composite	Heat Source	Target Cell	Application	Study Type	Additional Functions	Ref
**rGO@MSN**	NIR	A549	Cancer therapy	In vitro	Drug Delivery	[153]
**MrGO-AA-g-4-HC**	UV/Vis	MCF7 WS1	Chemo-photodynamic therapy Cancer therapy	In vitro/ In vivo	Drug Delivery	[141]
**rGO/MP-pyrene-PEG**	NIR	E. Coli UTI89 S. Aureus	Water disinfection Biotechnological	In vitro	Antibacterial	[43]
**rGO/MSN/PDA**	NIR	MHCC97-L MHCC97-H	Chemo-photothermal therapy	In vitro	Drug Delivery	[156]
**rGO-PDA**	NIR	HeLa	Antitumor therapy	In vitro/ *In vivo*	Drug Delivery	[157]
**ICG/CA-PPDN/rGO**	NIR	MDA-MB-231	Cancer therapy	In vitro/ In vivo	Bioimaging	[169]
**rGO/Co/PEG**	NIR AMF	L929 E. Coli	Antibacterial	In vitro	Antibacterial	[108]
**rGO-Ru-PEG**	UV/Vis NIR	A549	Cancer treatment	In vitro/ In vivo	Bioimaging	[170]
**GO-PEG-PEI-TPP@ICG**	NIR	MG63/DOX	Phototherapy	In vitro/ In vivo	Bioimaging	[171]
**GO-UCNP-Ce6**	NIR	L929 U14	Photodynamic/photothermal therapy	In vitro/ In vivo	Bioimaging	[167]
**GO/SBMA-PEI-PMAO**	NIR	MCF7 NHDF	Cancer photothermal therapy	In vitro	-	[172]
**GO-PEG**	NIR	CT26 HT-29	Cancer photothermal therapy	In vitro	-	[168]
**CuS(DOX)-GO-HA**	NIR	SCC-7 MDA-MB-231 BT-474	Cancer therapy	In vitro/ In vivo	Drug Delivery	[146]
**GO@Ge**	NIR	L929 MCF7	Chemo-photothermal therapy	In vitro	Drug Delivery	[148]
**MGO-PEG-CET**	NIR	CT26	Chemo-phototherapy	In vitro/ In vivo	Drug Delivery	[162]
**GO/Au-PEG-PLA**	NIR	MCF7 MDA HU02	Gene therapy	In vitro	Drug Delivery	[165]
**GO-PEG-PLA**	NIR	MCF7 MCF10A MDA-MB-231 HU02	Chemo-photothermal therapy	In vitro	Drug Delivery	[166]
**GO-CS-FA**	NIR	MDA-MB-231	Photothermal therapy	In vitro/ In vivo	Bioimaging	[173]
**PNIPAM/GO,** **PNIPAMAAM/GO**	NIR	MDA-MB-231	Chemo-photothermal therapy	In vitro	Drug Delivery	[174]

*Abbreviations: AA* Allylamine, *Au* Gold, *CA* Catechol, *Ce6* Chlorin e6, *CET* Cetuximab, *Co* Cobalt, *CS* Chitosan, *CuS* Copper sulfide, *DOX* Doxorubicin, *FA* Folic acid, *Ge* Gelatin, *GO* Graphene oxide, *HA* Hyaluronic acid, *HC* Hydroxycoumarin, *ICG* Indocyanine green, *MGO* Magnetic graphene oxide, *MP* Magnetic particle, *MrGO* Magnetic reduced graphene oxide, *MSN* Mesoporous silica nanoparticle, *PDA* Polydopamine, *PEG* Polyethylene glycol, *PEI* Polyethylenimine, *PLA* Poly-L-arginine, *PMAO* Poly(maleic anhydride-alt-1-octadecene), *PNIPAM* Poly(N-isopropylacrylamide), *PNIPAMAAM* Poly(N-isopropylacrylamide)-allylamine, *PPDN* Poly(ethylene glycol)-grafted poly(DMAEMA-co-NIPAAm), *rGO* Reduced graphene oxide, *Ru* Ruthenium, *SBMA* [2-(methacryloyloxy)ethyl]dimethyl-(3-sulfopropyl)ammonium hydroxide, *TPP* 4-Carboxybutyl)triphenyl phosphonium bromide, *UCNPs* Upconversion nanoparticles

Gulzar et al. [167] used both photodynamic and photothermal therapies against cancer cells by conjugating Chlorin e6 to upconversion nanoparticles that were then conjugated to GO. Singlet oxygen was generated alongside an increase in temperature under 808 nm irradiation which was successfully used *in *in vivo tumor treatment. The resulting upconversion luminescence was also used for imaging, making the particles a useful theranostic tool. Zhang et al. [170] followed the same strategy of using rGO nanosheets loaded with a PEG-modified Ru(II) complex (PEG-Ru) to target lysosomes in cancer cells for photodynamic and photothermal therapies, which were accomplished by applying 450 nm and 808 nm irradiation, respectively. Thermal-responsive release of the photosensitizer and the imaging agent PEG-Ru was also achieved.

### Bioimaging

Bioimaging generally uses natural fluorescence emission of GO and rGO, both in vivo and in vitro, for optical imaging, because both emit intense fluorescence at the appropriate excitation wavelength as shown in Table 3 [33, 34]. However, they have also been used for carrying contrast agents [175] for photoacoustic imaging [173, 176], MR imaging [177, 178], and single-photon emission computed tomography (SPECT) [177]. Additionally, GO has been used for Raman imaging [179]. While most research centers on cellular imaging, some groups have used GO for subcellular imaging of organelle-targeted cancer therapy [170, 171]. By tracking the GO/rGO nanoparticles, investigation of drug activation pathways involving cellular and organelle interactions can be achieved.

**Table 3:** List of graphene oxide and reduced graphene oxide composites and their bioimaging applications

Composite	Imaging Type	Target Cell	Study Type	Additional Functions	Ref
**ICG/CA-PPDN/rGO**	Fluorescence quenching Thermographic imaging	MDA-MB-231	In vitro/ In vivo	Phototherapy	[169]
**(CA-BODIPY)-PPDN/rGO**	Fluorescence quenching	MDCK MDA-MB-231	In vitro	Drug Delivery	[161]
**(CA–BODIPY)-PSMN/rGO**	Fluorescence quenching	-	*-*	-	[180]
**Gd-rGO**	Optical coherence tomography Fluorescence imaging	H1299	In vitro	Drug Delivery	[160]
**rGO-Ru-PEG**	Fluorescence imaging	A549	In vitro/ In vivo	Phototherapy	[170]
**rGO/Au**	Fluorescence imaging	Colo-205 MKN-45	In vitro	Biosensing	[35]
**Amine‐GO** **Sulfonate‐GO**	Fluorescence imaging	NIH-3T3 HeLa	In vitro	-	[34]
**GO@CP6 ⊃ PyN**	Photoacoustic imaging	U87MG	In vitro/ In vivo	-	[176]
**FA-CS-GO**	Photoacoustic imaging	MDA-MB-231	In vitro/ In vivo	Photothermal	[173]
**GO-M75**	Confocal Raman Microscopy	MDCK	In vitro	-	[179]

*Abbreviations: Au* Gold, *BODIPY* Boron-dipyrromethene, *CA* Catechol, *CP6 ⊃ PyN* Pillar[6]arene-based host–guest complex, *PPDN* Poly(ethylene glycol)-grafted poly(DMAEMA-co-NIPAAm), *FA* Folic Acid, *Gd* Gadolinium, *GO* Graphene oxide, *ICG* Indocyanine green, *PEG* Polyethylene glycol, *PPDN* PEG–g–poly (DMA–co–NIPAAm), *PSMN* Poly(sulfobetaine methacrylate-co-NIPAAm, *rGO* Reduced graphene oxide, *Ru* Ruthenium

Yogesh et al. [181] employed pure GO by incubating the cells with nanoparticles for 6 and 24 h and testing the fluorescence at two wavelengths, 405 and 488 nm, resulting in blue luminescence near the nuclear membrane and green luminescence at the 24-h mark. Mosaiab et al. [161] created a dual-responsive fluorescent GO nanoparticle that reacted to temperature and pH, where boron-dipyrromethene acted as the fluorescent dye, dimethylacetamide acted as the pH-responsive element, and N-isopropylacrylamide acted as the thermoresponsive element. The results indicated that a particle displayed fluorescence under lower pH and temperature (25 °C) and negligible fluorescence under physiological pH and temperature (37 °C) when excited with ultraviolet light at 365 nm. Qian et al. [177] designed a unique rGO-based nanoparticle capable of multimodal imaging combined with radioisotope therapy and chemotherapy for cancer theranostics. Manganese ferrite was grown *in situ* on the surface of rGO nanosheets and then functionalized with PEG. The resulting particle proved to be a good MR contrast agent, showing T1 and T2 weighted images. By labeling the nanocomposite with radionuclides ^125/131^I, SPECT was achieved alongside radioisotope therapy in conjunction with DOX loading for chemotherapy.

### Biosensing

Biosensors containing either GO or rGO generally exhibit fluorescence and fluorescence-quenching properties [35, 36]. However, high conductivity of rGO makes it useful for EC or ECL assays [37, 38]. Moreover, GO is often used in nucleotide detection owing to its strong interactions with ssDNA, allowing for detection of specific sequences [36, 182]. Although several applications involving GO/rGO in biosensing exist, those that uses of them as nanocarriers are limited [183, 184]. Applications of GO and rGO in which they were used in biosensors in the form of nanocarriers are listed in Table 4.

**Table 4:** List of graphene oxide and reduced graphene oxide composites and their biosensing applications

Composite	Application	Target Molecule	Method of Detection	Ref
**PAMMA-rGO**	Protein detection	Thrombin	ECL	[37]
**Arg/Au@Fe**_**3**_**O**_**4**_**–rGO**	Clinical diagnostics/ immunology	APE-1	ECL immunoassay	[38]
**rGO-Ca:CdSe**	Clinical diagnostics/ immunolgy	Prostate specific antigen	Photoelectrochemical immunoassay	[185]
**ABEI-PEI-PFO dots-rGOs/PtNPs**	Sensitive bioanalysis/ clinical	Kidney injury molecule-1	ECL immunoassay	[186]
**rGO/Au**	Cancer detection	L-Cysteine	Fluorescence sensing	[35]
**Dex-rGO**	Antiviral discovery screening	Dengue virus	Fluorescence quenching and recovery	[182]
**GO-PEGMA**	Noninvasive detection/targeting	DNA Thrombin Adenosine miR-10b	Fluorescence quenching	[187]

*Abbreviation: ABEI* N-(aminobutyl)-N-(ethylisoluminol), *APE* Apurinic/apyrimidinic endonuclease 1, *Arg* Arginine, *Au* Gold, *Ca* Calcium, *CdSe* Cadmium selenide, *Dex* Dextran, *Fe*_*3*_*O*_*4*_ Iron oxide, *GO* Graphene oxide, Poly(9,9-dioctylfluorenyl-2,7-diyl), *PAMMA* Polyamidoamine, *PEGMA* Polyethylene glycol methyl-ether-methacrylate, *PEI* Polyethylenimine, *PFO* Poly(9,9-dioctylfluorenyl-2,7-diyl), *PtNPs* Platinum nanoparticles, *rGO* Reduced graphene oxide

Xia et al. [36] detected single nucleotide polymorphisms (SNPs) by embedding them in SYBR Green I (SG) before adding GO particles. Subsequently, fluorescence from SG in unstable SNPs was highly quenched by GO within 3 min, whereas fluorescence from perfectly complementary dsDNA was comparatively high. Yuan et al. [37] designed a pseudobienzyme aptasensor with polyamidoamine-rGO as a nanocarrier conjugated with a hemin/G-quadruplex as nicotinamide adenine dinucleotide oxidase and horseradish peroxidase-mimicking DNA enzyme to detect thrombin. Cyclic voltammetry and differential pulse voltammetry revealed that the particle was capable of highly sensitive and selective detection of thrombin.

### Tissue engineering

Both GO and rGO encourage stem cell proliferation and differentiation while functioning as scaffolds or parts of a scaffold, making them ideal for tissue engineering and regeneration, the various applications of which are listed in Table 5. While GO and rGO nanoparticles can be incorporated into scaffolds for general tissue engineering, such as skin [188–190], cartilage [191, 192], bone [189, 193–195], and muscle tissue [196, 197] rGO is commonly incorporated into nanofibers to enhance their electroconductivity, which is a significant factor in cardiac and nerve tissue regeneration [39–41]. Tissue engineering applications can also implement GO/rGO for morphological [198] and photoelectric [199] stimulations to encourage cell proliferation/differentiation.

**Table 5:** List of graphene oxide and reduced graphene oxide composites and their tissue engineering applications

Composite	Scaffold Type	Tissue	Cell	Study Type	Ref
**SF/rGO**	Nanofibers	Neuronal	NG108-15	In vitro	[39]
**SF/RGO, SF/GO**	Nanofibers	General	Schwann cells	In vitro	[40]
**rGO/GelMA/PCL**	Nanofibers	Neuronal	RSC96	In vitro/ In vivo	[41]
**PVPA–ESM/rGO**	Nanofibers	Skin	PC-12	In vitro	[188]
**PCL/rGO**	Nanofibers	Bone	MG-63	In vitro	[189]
**PVA/rGO**	Nanofibers	Skin	CCD-986Sk	In vitro	[190]
**RGO-AuNPs@PCL**	Nanofibers	Neuronal	S42 PC-12	In vitro	[200]
**Amine-rGO@Alg/ECM**	Hydrogel	Cardiac	HUVEC	In vitro	[201]
**ECM-rGO**	Hydrogel	Cardiac	hiPSC-CM HS-27A	In vitro	[202]
**Ge/MV/GO**	Hydrogel	Bone Vascular	BMSC	In vitro/ In vivo	[193]
**SPION-rGO/Collagen**	Hydrogel	Neuronal	SH-SY5Y	In vitro	[198]
**rGO/g-C**_**3**_**N**_**4**_**/TiO**_**2**_	Nanocoating	Neuronal Bone	MC3T3-E1 PC-12	In vitro	[199]
**PLA/GO-CS**	Porous scaffold	General	L929	In vitro	[203]
**GG/PEGDA/GO**	Hydrogel	Cartilage	OA chondrocytes	In vitro	[191]
**GO-HY**	Gel	Bone	MC3T3-E1	In vitro/ In vivo	[194]
**Alg/Ser/GO**	Hydrogel	Bone	Raw 264.7 BMSC	In vitro/ In vivo	[195]
**PU-GO**	Nanofibers	Skeletal muscle	C2C12	In vitro	[196]
**Ca-Alg/PCL/rGO**	Hydrogel	Skeletal muscle	C2C12	In vitro	[197]
**GO/PLGA**	Nanofibers	General	hMSCs	In vitro	[204]

*Abbreviations: Alg* Alginate, *AuNPs* Gold nanoparticles, *Ca* Calcium, *CS* Chitosan, *ECM* Extracellular matrix, *ESM* Egg shell membrane, *g-C*_*3*_*N*_*4*_ Graphitic-carbon nitride, *Ge* Gelatin, *GelMA* Gelatin methacryloyl, *GG* Gellan gum, *GO* Graphene oxide, *HY* Sodium hyaluronate, *MV* Methyl vanillate, *PCL* Polycaprolactone, *PEGDA* Polyethylene glycol diacrylate, *PLGA* Poly(lactic-co-glycolic acid), *PU* Polyurethane, *PVA* Polyvinyl alcohol, *PVPA* Polyvinylpyrrolidone-acrylic acid hydrogel, *rGO* Reduced graphene oxide, *Ser* Sericin, *SF* Silk fibroin, *SPION* Superoxide paramagnetic iron oxide nanoparticle, *TiO*_*2*_ Titanium dioxide

Wang et al. [204] incorporated polyethylenimine-modified GO into an electrospun poly(D,L-lactic-co-glycolic acid) scaffold, which was then loaded with plasmid DNA (pDNA) to improve the growth and differentiation of mesenchymal stem cells via solid-phase gene delivery. Loading the nanofibers with pDNA nearly doubled the transfection efficiency compared to simply mixing it into the medium, improving it from 12.1% to 23.6%. Fang et al. [41] created an electrospun gelatin methacryloyl/polycaprolactone scaffold with rGO interspersed throughout to act as a nerve guidance conduit. The addition of low concentrations of rGO (0.25 and 0.5 wt%) increased the electroconductivity of the scaffold and improved nerve tissue regeneration.

### Antibacterial applications

Both GO and rGO possess antibacterial properties that are ideal for antibacterial applications, as listed in Table 6. Nanocarriers are generally directly applied to bacteria-containing media at high concentrations [42, 43] or incorporated into a membrane [205, 206]. The cells were inactivated as GO/rGO nanosheets aggregated and caused oxidative stress [96, 106]. They can also be used in conjunction with photothermal therapy for cell ablation, increasing its effectiveness [43, 108].

**Table 6:** List of graphene oxide and reduced graphene oxide composites and their antibacterial applications

Composite	Host Material	Target Cells	Additional Functions	Ref
**rGO/MP-pyrene-PEG**	-	E. coli S. aureus	Phototherapy	[43]
**rGO-CUR**	-	E. faecalis	Phototherapy	[207]
**PCL/rGO-Ag**	Fibrous membrane	E. coli S. aureus	-	[205]
**Zn-dopamine-rGO**	-	T-47D MCF10A	Drug delivery	[159]
**rGO/Co/PEG**	-	L929 E. coli	Phototherapy	[108]
**ZIF-8/GO**	-	E. coli S. aureus	-	[208]
**GO/AgNP, GO/CuNP**	-	E. coli S. aureus	-	[42]
**GO/NiO/starch**	-	S. aureus	-	[209]
**GO/PMMA**	Nanofiber	E. coli	-	[210]
**PVA/Ag/GO-IL**	Film	E. coli S. aureus	-	[206]

*Abbreviations: Ag* Silver, *AgNp* Silver nanoparticle, *Co* Cobalt, *CuNp* Copper nanoparticle, *CUR* Curcumin, *GO* Graphene oxide, *IL* Ionic liquid, *MP* Magnetic particle, *NiO* Nickel oxide, *PCL* Polycaprolactone, *PEG* Polyethylene glycol, *PMMA* Polymethyl methacrylate, *PVA* Polyvinyl alcohol, *rGO* Reduced graphene oxide, *ZIF-8* Zeolitic imidazolate framework 8, *Zn* Zinc

Halouane et al. [43] created rGO particles conjugated with nitrodopamine-coated magnetic nanoparticles (MP_ND_) and pyrene-PEG with antifimbrial antibodies immobilized on the surface. The MP_ND_ served to capture the pathogens, and NIR irradiation at a wavelength of 980 nm ablated the captured pathogens at temperatures up to 75 °C. Matharu et al. [210] generated poly(methyl 2-methylpropenoate) fiber meshes with dispersed GO nanosheets. Fibers with 8 wt% concentration of GO were most effective at bacterial reduction, with killing efficacy reaching 85 ± 1.4%, with the cytotoxic mechanism being attributed to the production of oxidative stress.

## Conclusion

In summary, GO exhibits excellent properties suitable for various biomedical applications, including a high colloidal stability, good biocompatibility, and antibacterial properties. In particular, GO can be a good nanocarrier because it can be conjugated, embedded, or loaded with drugs, proteins, metals, and biomolecules. Moreover, it can be reduced to obtain highly conductive rGO at the expense of colloidal stability, which may be beneficial for biosensors and tissue engineering. In this review, we discuss the properties of GO and rGO, and the potential methods of functionalization with polymers and other molecules through covalent and noncovalent bonding. Through functionalization, GO and rGO have been engineered to be specific and functional nanocarriers of therapeutic biomolecules, such as anticancer drugs and genes, or modified for phototherapy, bioimaging, biosensing, tissue engineering, and antibacterial applications.

Despite the good biocompatibility of GO and rGO at lower concentrations, several mechanisms that may induce cytotoxicity and genotoxicity have been identified, including the aggregation of cell membrane damage and oxidative stress. Notably, there was a difference in cytotoxicity between mammalian cells and bacteria, where an increase in GO/rGO nanosheet size increased cytotoxicity in both, but the effect was more significant in bacteria. However, the comparison did not take into account the inherent size difference between mammalian cells and bacteria, and therefore, the relative size of the GO/rGO sheets as compared to the cells. Future studies should consider the cytotoxicity of GO/rGO as a function of the nanoparticle-to-cell size ratio. Furthermore, GO and rGO tended to aggregate in certain organs, even when functionalized. This is a cause for concern because while in vivo studies have deemed the use of these nanoparticles to be generally non-lethal, some toxic effects have been identified. With the long-term effects remaining largely unexplored, it is currently difficult to extend the use of GO/rGO as *an *in vivo nanocarrier in clinical trials. As such, GO- and rGO-based nanocomposite nanocarriers have great potential in biomedical applications; however, further studies on their effects in vivo are still necessary to advance the field.

## Data Availability

Not applicable.

## Abbreviations

2D: Two-dimensional
Ag: Silver
CPT: Camptothecin
CS: Chitosan
DOX: Doxorubicin
dsDNA: Double-stranded DNA
EC: Electrochemical
ECL: Electrochemiluminescence
Fa: Folic acid
GO: Graphene oxide
H_2_SO_4_: Sulfuric acid
HC: 4-Hydroxycoumarin
HNO_3_: Nitric acid
IONP: Iron oxide hybrid nanocomposite
KClO_3_: Potassium chlorate
LbL: Layer-by-layer
MPND: Magnetic nanoparticles
MR: Magnetic resonance
NIR: Near-infrared
PAC: Paclitaxel
pDNA: Plasmid DNA
PEG: Polyethylene glycol
PLL: Poly-L-lysine
PSA: Polysebacic anhydride
R8: Octaarginine
rGO: Reduced graphene oxide
SG: SYBR Green I
siRNA: Small interfering RNA
SNP: Single nucleotide polymorphism
SPECT: Single-photon emission computed tomography
ssDNA: Single-stranded DNA
